# Early pregnancy vitamin D and the risk of adverse maternal and infant outcomes: a retrospective cohort study

**DOI:** 10.1186/s12884-020-03158-6

**Published:** 2020-08-14

**Authors:** Geng-dong Chen, Ting-ting Pang, Peng-sheng Li, Zi-xing Zhou, Dong-xin Lin, Da-zhi Fan, Xiao-ling Guo, Zheng-ping Liu

**Affiliations:** 1 Foshan Institute of Fetal Medicine, Department of Obstetrics, Affiliated Foshan Maternity & Child Healthcare Hospital, Southern Medical University, Foshan, 528000 Guangdong China; 2 Department of Medical Records, Affiliated Foshan Maternity & Child Healthcare Hospital, Southern Medical University, Foshan, 528000 Guangdong China

**Keywords:** 25(oh)d, Vitamin D, Pregnancy, Maternal outcome, Infant outcome

## Abstract

**Background:**

Previous evidence has suggested that lower gestational vitamin D levels might increase the risks of adverse pregnancy and birth outcomes. The results remain inconsistent and require further exploration.

**Methods:**

A total of 2814 Chinese mother-infant pairs were included in this retrospective cohort study. Serum concentrations of 25(OH)D were reviewed in early pregnancy (16.3 ± 2.3 weeks). Outcomes of maternal gestational diabetes mellitus (GDM), cesarean section, fetal distress, preterm birth, low birth weight (LBW), and macrosomia were extracted from the medical records. Cox regression analysis was used to explore these associations.

**Results:**

In total, 19.3% of mothers were pregnant at an advanced age (≥35 years), and 40.3% of pregnant women had vitamin D deficiency (< 50 nmol/L). After adjusting for potential covariates, the hazard ratio (*HR) (95% CI)* per standard deviation (SD) increase of serum 25(OH)D concentrations was 0.86 (0.779, 0.951) for GDM, 0.844 (0.730, 0.976) for preterm birth, and 0.849 (0.726, 0.993) for LBW. Similar protective associations were found for GDM, cesarean section, and preterm birth for a better vitamin D status when compared with vitamin D deficiency.

**Conclusion:**

Higher early pregnancy vitamin D was associated with a lower risk of GDM, cesarean section, preterm birth, and LBW.

## Background

Vitamin D is a secosteroid hormone that is well known for its physiological function in maintaining bone metabolism and health. A high prevalence of vitamin D deficiency (usually defined as serum 25(OH)D levels < 50 nmol/L) has been found in pregnant women globally, especially in developing countries [1], including China [2, 3]. Increasing evidence has suggested that vitamin D sufficiency is important for the prevention of pregnancy complications in mothers and adverse fetal birth outcomes [4, 5], although divergent results have been reported in other studies [3, 6–8]. Several systematic reviews based on randomized clinical trials or observational studies have suggested that lower vitamin D status contribute to adverse outcomes such as preeclampsia [9], gestational diabetes mellitus (GDM) [10, 11], low birth weight (LBW) [12], and preterm birth [13]. However, increasing evidence shows different associations [3, 7, 14], and no definitive conclusion has yet been made [4]. Several problems remain to be solved by further studies: the heterogeneity of associations from diverse areas and populations with different vitamin D status serve as evidence when making guidelines for specific regions; the observation or supplementation of vitamin D in the third trimester in many studies might present the problem of causal inference or miss the practical period for the intervention. In addition, the results from early trimesters might be helpful and more important for the early prevention of adverse outcomes.

In addition, it has been suggested that the risk of adverse complications or birth outcomes increases as maternal age increases [15–17]. With the change of population policy in China, the percentage of women pregnant at an advanced age (> 35 years) has increased, and proper strategies are urgently needed for the prevention of adverse complications [18]. However, whether the influence of vitamin D on maternal and infant outcomes remained the same for women pregnant at young (< 35 years) and advanced ages remains unclear, and more studies are needed to better illustrate the problem.

We investigated the relationship between early gestational serum 25(OH)D concentrations and several adverse maternal and infant outcomes in a retrospective cohort study including 2814 Chinese mother-infant pairs. Our results provide further evidence for clinical recommendations on the early prevention of related adverse outcomes in this field.

## Methods

The study used data that were gathered from a large center, Affiliated Foshan Maternity & Child Healthcare Hospital, Southern Medical University, Foshan City, Guangdong Province, China, from September 1, 2017, to July 31, 2018. The hospital is the largest gynecology and obstetrics center in Foshan City and covers a large population of 7.7 million people. The included subjects were women who underwent early pregnancy serum vitamin D measurement (≤20 gestational weeks) and delivered their infants at the hospital. The exclusion criteria included twin or higher-order multiple pregnancies; serious diseases, such as type 2 diabetes mellitus, cardiovascular diseases, thyroid disorder, and cancer, that occurred before pregnancy. Ultimately, a total 2814 mother-infant pairs were included in this study. The study was approved by the ethics committee of Affiliated Foshan Maternity & Child Healthcare Hospital, Southern Medical University.

### Data collection

Vitamin D data from the clinical laboratory were reviewed. Blood samples were collected during the regular obstetric check-ups and immediately measured by a clinical laboratory without being frozen. Serum concentrations of 25(OH)D, 25(OH)D_2_, and 25(OH)D_3_ were detected using colloidal gold immunochromatography. Commercial kits were obtained from Mei Ning Kang Cheng Bio Tec Inc. The intra-assay and inter-assay coefficients of variation were less than 15%.

Outcomes including GDM, cesarean section, fetal distress, preterm birth, low birth weight, and macrosomia were extracted from medical records and reexamined by two independent staff members. The disease diagnoses were made by professional doctors with the same standardization criteria and were extracted from the medical records. Gestational hypertension, preeclampsia, or eclampsia was not included because of the lack of available cases. An oral glucose tolerance test was performed from 24 to 28 gestational weeks, and GDM was diagnosed if the subjects met any of the following criteria: fasting blood glucose ≥5.1 mmol/L; one-hour blood glucose postoral sugar ≥10.0 mmol/L; or two-hour blood glucose postoral sugar ≥8.5 mmol/L. Preterm birth was defined as delivery at ≥28 but < 37 gestational weeks. LBW was diagnosed if the neonatal birth weight < 2500 g, while a neonatal birth weight ≥ 4000 g was defined as macrosomia. Other variables, including the maternal age, body mass index (BMI), gestational age, parity, season of blood collection, and time of delivery, were also extracted from the medical records.

### Statistical analyses

Continuous variables were represented by the mean ± standard deviation (SD) or median (interquartile range) and tested by Student’s t-test. Categorical variables were represented by frequencies (percentage) and tested by the chi-square test. Cox regression analysis was performed to explore the associations between vitamin D and maternal or infant outcomes. Serum concentrations of 25(OH)D, 25(OH)D_2_, and 25(OH)D_3_ were first Z-standardized before being included in the regression. Vitamin D deficiency was defined as serum 25(OH)D levels of < 50 nmol/L. Two different models were tested, with Model 1 as a univariate model without adjustment and Model 2 adjusted for maternal age, BMI, parity, and season the blood was collected and measured. Stratified analyses were performed according to the gestational age (young: < 35 years; advanced: ≥35 years). All the analyses were performed using SPSS software version 21.0 (SPSS Inc. Chicago, IL, USA). A two-sided *P* value of less than 0.05 was considered statistically significant.

## Results

A total 2814 mother-infant pairs were included in this study. A high prevalence of gestational vitamin D deficiency (40.3%) was discovered in the mothers. Even in subjects without vitamin D deficiency, the highest serum 25(OH)D concentration was only 92.2 nmol/L. Compared with women pregnant at a younger age, the subjects with advanced age of pregnancy (≥35 years) tended to have a higher BMI, parity, higher percentage of vitamin D deficiency, higher incidence of GDM, cesarean section, preterm birth, and LBW, but a lower gestational age, lower serum 25(OH)D and 25 (OH)D_3_ concentrations, lower incidence of fetal distress, and lower incidence of macrosomia (Table 1).

**Table 1:** Characteristic of subjects

	Total (*N* = 2814)	< 35 years (*N* = 2272)	≥35 years (*N* = 542)	*P*
Age, years	30.5 ± 4.98	28.7 ± 3.47	38.2 ± 2.30	< 0.001
BMI, kg/cm^2^	26.6 ± 3.12	26.5 ± 3.11	27.4 ± 3.04	< 0.001
Gestational age, weeks	38.7 ± 1.84	38.8 ± 1.80	38.2 ± 1.98	< 0.001
Parity, times	1.45 ± 0.56	1.35 ± 0.53	1.88 ± 0.44	< 0.001
Neonatal birth weight, kg	3.15 ± 0.45	3.15 ± 0.44	3.14 ± 0.46	0.654
25(OH)D, nmol/L	53.1 ± 9.99	53.5 ± 9.98	51.9 ± 9.89	0.002
25(OH)D_2_, nmol/L	5.34 ± 1.71	5.34 ± 1.00	5.33 ± 3.32	0.884
25(OH)D_3_, nmol/L	47.8 ± 8.99	48.0 ± 8.98	46.7 ± 8.95	0.002
Vitamin D deficiency, N (%)				0.043
Yes	1133 (40.3)	894 (39.3)	239 (44.1)	
No	1681 (59.7)	1378 (60.7)	303 (55.9)	
Gestational diabetes mellitus, N (%)				< 0.001
Yes	424 (15.1)	279 (12.3)	145 (26.8)	
No	2390 (84.9)	1993 (87.7)	397 (73.2)	
Caesarean section, N (%)				< 0.001
Yes	1368 (48.6)	1021 (44.9)	347 (64.0)	
No	1446 (51.4)	1251 (55.1)	195 (36.0)	
Fetal distress in uterus, N (%)				0.001
Yes	282 (10.0)	249 (11.0)	33 (6.1)	
No	2532 (90.0)	2023 (89.0)	509 (93.9)	
Preterm birth, N (%)				< 0.001
Yes	195 (6.90)	135 (5.90)	60 (11.1)	
No	2619 (93.1)	2137 (94.1)	482 (88.9)	
Low birth weight, N (%)				0.025
Yes	166 (5.90)	123 (5.40)	43 (7.90)	
No	2648 (94.1)	2149 (94.6)	499 (92.1)	
Macrosomia, N (%)				0.030
Yes	74 (2.60)	67 (2.90)	7 (1.30)	
No	2740 (97.4)	2205 (97.1)	535 (98.7)	

As shown in Table 2, although the 25(OH)D concentrations were statistically higher in spring and summer than those in autumn and winter, only small difference of 25(OH)D values (0.39 to 3.2 nmol/L) was observed between different seasons, especially for 25(OH)D_2_. Significant protective associations were found between the vitamin D levels and GDM and preterm birth. After adjusting for potential covariates (Table 3), higher 25(OH)D concentrations (per one SD increase) were associated with a 13.9% (*HR*: 0.861, *95% CI*: 0.779, 0.951) decrease in the GDM risk, a 15.6% (*HR*: 0.844, *95% CI*: 0.730, 0.976) decrease in the preterm birth risk, and a 15.1% (*HR*: 0.849, *95% CI*: 0.726, 0.993) decrease in the LBW risk. Similar protective results were also found for 25(OH)D_2_ and 25(OH)D_3_, but the associations tended to be more pronounced for 25(OH)D_2_. Null associations between vitamin D and cesarean section, fetal distress, and macrosomia were observed in all the subjects. Maternal serum 25(OH)D levels ≥50 nmol/L were associated with a 25.0% decrease in the GDM risk, a 15.2% decrease in the cesarean section risk, and a 26.8% decrease in the preterm birth risk, but the trend did not hold with the other outcomes, compared with those with vitamin D deficiency (Table 4).

**Table 2:** Seasonal difference between serum vitamin D indicators

	Detected Season		*P*
	spring & summer	autumn & winter	
25(OH)D, nmol/L	55.2 ± 10.2	52.0 ± 9.68	< 0.001
25(OH)D_2_, nmol/L	5.59 ± 2.53	5.20 ± 0.97	< 0.001
25(OH)D_3_, nmol/L	49.6 ± 9.19	46.7 ± 8.71	< 0.001

**Table 3:** Associations between early pregnant serum vitamin D concentrations and maternal & infant outcomes

	25(OH)D ^a^			25(OH)D_2_^a^			25(OH)D_3_^a^		
	*HR*	*95%CI*	*P*	*HR*	*95%CI*	*P*	*HR*	*95%CI*	*P*
Gestational diabetes mellitus									
Model 1	**0.832**	**(0.754, 0.919)**	**< 0.001**	**0.734**	**(0.621, 0.867)**	**< 0.001**	**0.833**	**(0.754, 0.919)**	**< 0.001**
Model 2	**0.861**	**(0.779, 0.951)**	**0.003**	**0.776**	**(0.656, 0.918)**	**0.003**	**0.861**	**(0.779, 0.952)**	**0.003**
Caesarean section									
Model 1	**0.923**	**(0.873, 0.975)**	**0.004**	0.954	(0.883, 1.032)	0.243	**0.923**	**(0.873, 0.975)**	**0.004**
Model 2	0.957	(0.905, 1.012)	0.122	0.981	(0.929, 1.035)	0.480	0.957	(0.905, 1.012)	0.120
Fetal distress									
Model 1	0.931	(0.824, 1.051)	0.246	0.885	(0.723, 1.084)	0.239	0.930	(0.824, 1.050)	0.244
Model 2	0.941	(0.833, 1.064)	0.333	0.903	(0.736, 1.107)	0.326	0.941	(0.833, 1.064)	0.333
Preterm birth									
Model 1	**0.840**	**(0.727, 0.970)**	**0.017**	**0.745**	**(0.585, 0.950)**	**0.017**	**0.839**	**(0.727, 0.969)**	**0.017**
Model 2	**0.844**	**(0.730, 0.976)**	**0.022**	**0.752**	**(0.589, 0.959)**	**0.022**	**0.844**	**(0.730, 0.975)**	**0.021**
Low birth weight									
Model 1	0.866	(0.741, 1.012)	0.070	0.785	(0.604, 1.020)	0.070	0.866	(0.741, 1.012)	0.069
Model 2	**0.849**	**(0.726, 0.993)**	**0.041**	**0.759**	**(0.583, 0.988)**	**0.041**	**0.849**	**(0.726, 0.993)**	**0.040**
Macrosomia									
Model 1	1.078	(0.849, 1.369)	0.538	1.007	(0.857, 1.185)	0.930	1.077	(0.848, 1.368)	0.541
Model 2	1.148	(0.893, 1.475)	0.281	1.031	(0.900, 1.182)	0.657	1.147	(0.893, 1.475)	0.282

Cox regression analysis were operated for exploration of associations. Model 1: without adjustment. Model 2: adjusted for age, BMI, parity, season of blood collected. a: Per one SD increase

**Table 4:** Associations between vitamin D status and maternal & infant outcomes

	25(OH)D			
		*HR*	*95%CI*	*P*
Gestational diabetes mellitus	< 50 nmol/L	1.000		
	≥50 nmol/L	**0.750**	**(0.618, 0.909)**	**0.003**
Caesarean section	< 50 nmol/L	1.000		
	≥50 nmol/L	**0.848**	**(0.761, 0.945)**	**0.003**
Fetal distress	< 50 nmol/L	1.000		
	≥50 nmol/L	0.807	(0.636, 1.023)	0.076
Preterm birth	< 50 nmol/L	1.000		
	≥50 nmol/L	**0.732**	**(0.551, 0.972)**	**0.031**
Low birth weight	< 50 nmol/L	1.000		
	≥50 nmol/L	0.747	(0.549, 1.016)	0.063
Macrosomia	< 50 nmol/L	1.000		
	≥50 nmol/L	0.967	(0.595, 1.570)	0.892

Cox regression analysis were operated for exploration of associations, and adjusted for covariates including: age, BMI, parity, season of blood collected

After stratification by gestational age (Table 5), higher levels of vitamin D were associated with a 15.9% lower risk of GDM in those < 35 years. In contrast, a protective association of vitamin D with low birth weight was found for women pregnant at ≥35 years but not < 35 years. However, no significant interactions were discovered (*P*-interaction = 0.095 ~ 0.986).

**Table 5:** Subclass analysis of relationship between serum vitamin D concentrations and related outcomes stratified by gestational age

		25(OH)D ^a^			*P* ^b^	25(OH)D_2_^a^			*P* ^b^	25(OH)D_3_^a^			*P* ^b^
		*HR*	*95%CI*	*P*		*HR*	*95%CI*	*P*		*HR*	*95%CI*	*P*	
Gestational diabetes mellitus	< 35 y	**0.841**	**(0.744, 0.951)**	**0.006**	0.744	**0.748**	**(0.608, 0.919)**	**0.006**	0.751	**0.842**	**(0.745, 0.952)**	**0.006**	0.749
	≥35 y	0.900	(0.755, 1.072)	0.238		0.834	(0.622, 1.119)	0.227		0.900	(0.755, 1.072)	0.238	
Caesarean section	< 35 y	0.970	(0.909, 1.035)	0.354	0.461	0.950	(0.852, 1.059)	0.353	0.484	0.970	(0.909, 1.034)	0.348	0.466
	≥35 y	0.939	(0.839, 1.052)	0.281		0.995	(0.947, 1.046)	0.850		0.940	(0.839, 1.053)	0.282	
Fetal distress	< 35 y	0.941	(0.826, 1.072)	0.362	0.983	0.903	(0.724, 1.125)	0.363	0.972	0.941	(0.825, 1.072)	0.360	0.986
	≥35 y	0.924	(0.642, 1.330)	0.672		0.872	(0.477, 1.593)	0.656		0.925	(0.643, 1.331)	0.675	
Preterm birth	< 35 y	0.886	(0.745, 1.054)	0.172	0.264	0.816	(0.610, 1.093)	0.173	0.263	0.886	(0.745, 1.054)	0.171	0.264
	≥35 y	0.773	(0.594, 1.007)	0.056		0.648	(0.416, 1.009)	0.055		0.773	(0.594, 1.006)	0.056	
Low birth weight	< 35 y	0.911	(0.760, 1.093)	0.317	0.145	0.855	(0.629, 1.162)	0.317	0.145	0.930	(0.779, 1.110)	0.423	0.145
	≥35 y	**0.721**	**(0.528, 0.984)**	**0.039**		**0.575**	**(0.341, 0.972)**	**0.039**		**0.721**	**(0.528, 0.984)**	**0.039**	
Macrosomia	< 35 y	1.139	(0.876, 1.480)	0.331	0.784	1.245	(0.801, 1.935)	0.331	0.425	1.138	(0.876, 1.479)	0.333	0.784
	≥35 y	1.332	(0.511, 3.476)	0.558		1.034	(0.724, 1.477)	0.853		1.331	(0.510, 3.474)	0.559	

Cox regression analysis were operated for exploration of associations, and adjusted for covariates including: age, BMI, parity, season of blood collected

^a^: Per one *SD* increase. ^b^: *P* for interaction

## Discussion

In this retrospective cohort study including 2814 Chinese mother-infant pairs, protective associations were found for higher serum 25(OH)D concentrations with GDM, cesarean section, postpartum hemorrhage, preterm birth, and low birth weight, but not for the other outcomes. The results for 25(OH)D_2_ tended to be more pronounced than those for 25(OH)D_3_. Interestingly, higher serum vitamin D contributes to a higher risk of postpartum anemia in women pregnant at a young age but not in advanced years.

In this population with a high prevalence of vitamin D deficiency, better serum vitamin D status contributed to a lower risk of GDM. This result was consistent with several other studies. A 49 to 85% higher risk of GDM was observed for pregnant women with insufficient or deficient vitamin D status in three systematic reviews and meta-analyses based on observational studies or clinical trials [10, 12, 19]. In a large randomized control trial (RCT) based on 800 Iranian women, the intervention of 25(OH)D_3_ was associated with lower risk of GDM [20]. The results were further supported by several other prospective cohort studies [21, 22]. However, null associations were found between the vitamin D status or supplements and GDM in a systematic review based on five randomized trials including 1030 subjects [7], a nested case-control study of 5109 women [23], and a large prospective study including 2382 mother-child pairs [14]. Additionally, two studies found detrimental associations of higher vitamin D levels with GDM, but the effects were tiny (1.7%) [3] or restricted to specific ethnicities (Hispanic) [6].

Consistent with our results, the protective association of maternal vitamin D status with a lower risk of preterm or low birth weight has also been reported in several studies. An inverse dose-response relation of vitamin D status was found for preterm birth in a systematic review and meta-analysis based on longitudinal studies [24]. Rostami et al. reported that a 25(OH)D_3_ intervention of monthly 50,000 IU could be beneficial for the prevention of preterm delivery in an RCT of 800 Iranian women [20]. In a large prospective cohort including 3658 mother-offspring pairs, the risk of low birth weight was 12.3 (95% CI: 4.47, 33.89) and 3.15 (95% CI: 1.06, 9.39) among subjects with vitamin D deficiency and insufficiency, respectively [25]. Higher maternal serum 25(OH)D was positively associated with higher birth weight in a study of United Arab Emirates [26], and supplementation of vitamin D at a dose of 4000 IU/d was optimal and safe for mothers and their infants in United Arab Emirates [27]. However, null or detrimental associations were also reported in other studies. Although vitamin D increased the mean birth weight, it did not significantly reduce the risk of low birth weight or preterm birth in a meta-analysis based on randomized trials [28]; these results were further supported by another update study [7]. In addition, higher 25(OH)D concentrations were found to increase the 3.9% risk of preterm delivery in a prospective study of 2960 pregnant Chinese women [3].

Gestational vitamin D deficiency was found to be associated with a 2-fold increased risk of cesarean section in a cohort of 1153 low-income pregnant women [29]. Within a cohort of 253 women from the United States, for women with 25(OH)D concentrations lower than 37.5 nmol/L, the risk of a primary cesarean section was almost 3.84 times higher [30]. These results were consistent with ours. However, a meta-analysis of 17 trials (3240 subjects) found null associations of vitamin D supplements with cesarean section [7].

Much heterogeneity exists in the previous evidence in this field, and there may be several factors that partly explain these differences. First, most of the randomized trials included had a small sample size and were of low quality. Most subjects included in the trials had sufficient vitamin D status (≥75 nmol/L), and a further dose of a 400 or 600 IU/d supplement might have attenuated the detrimental influence of vitamin D deficiency or insufficiency in the reference group [7]. Increasing benefits might not exist for higher doses. Second, the vitamin D receptor is important for the utilization of vitamin D [31, 32]. The difference in vitamin D receptor gene polymorphism might help explain the ethnic heterogeneity [33]. This requires further confirmation in the future. Third, the studies were conducted during different trimesters of gestation, which might increase the difficulty of making comparisons; those studies conducted during an early gestational period might be advantageous for causal inference and early prevention.

Because 25(OH)D_3_ contributes most to the 25(OH)D concentrations, it has been used to represent 25(OH)D in many studies, and only a few studies have focused on 25(OH)D_2_. In our study, the results were generally consistent between these two subcomponents. The associations tend to be more pronounced in 25(OH)D_2_ than in 25(OH)D_3_ and might indicate the prominence of 25(OH)D_2_ over 25(OH)D_3_ for the prevention of related diseases. This theory merits further confirmation. Nevertheless, our results and others provide further evidence for the importance of a better early vitamin D status for the prevention of GDM, cesarean section, preterm birth, and low birth weight in a population with a high prevalence of vitamin D deficiency. Our results indicated that more consideration should be given to distinguishing between the vitamin D subcomponents, women pregnant at different ages, and women with more pregnancy complications in this field. Considering the high prevalence of vitamin D deficiency during pregnancy observed in China [2, 3], it is a matter of urgency to call for the supplementation of vitamin D, and more efforts should be made to improve the gestational vitamin D status of Chinese population.

Vitamin D deficiency was found to decrease vascular diameter within the labyrinth region and dysregulate placental development during early pregnancy in an animal experiment [34]. Vitamin D supplementation during early pregnancy might rescue this situation, while further supplementation might be futile after missing this time point [35]. Moreover, vitamin D supplementation during early pregnancy is likely to affect genetic information of systemic inflammation and immune responses involved in the development of gestational comorbidities (e.g., GDM, preeclampsia, and infection) as reviewed by Hollis et al [36] These mechanisms help to explain the beneficial influences of vitamin D for maternal and infant health and emphasize the importance of improving vitamin D status during early pregnancy.

Our study had several advantages. First, serum 25(OH)D concentrations were measured during an early gestational period, and with the retrospective cohort design, we assured the temporal sequence and avoided the possibility of causal inversion. Second, we studied multiple outcomes and conducted further analysis of the sub-component of vitamin D and a stratified analysis of gestational age, which provided a more comprehensive understanding of this field. There are also several limitations of our study. First, our study was limited by the lack of information on dietary vitamin D intake (including by supplement) and sunlight exposure during the pregnancy period. The obtained data were difficult to use for a retrospective study, while the use of blood indicators might be more precise than a dietary survey, so we adjusted the association for different seasons to attenuate their influence. Second, the 25(OH)D concentration was measured only once; we could not monitor the dynamic changes afterward or determine whether they were normalized after receiving vitamin D supplementation. However, the associations tended to be underestimated instead of overestimated. Third, Wagner et al. [37] and Mirzakhani et al. [35] indicated a 25(OH)D concentration ≥ 100 nmol/L in early pregnancy was optimal for the prevention of preterm birth and preeclampsia; however, the highest 25(OH)D concentration in our study is only 92.2 nmol/L. Therefore, we were unable to perform the analyses using a threshold of 100 nmol/L and assess the influences of higher 25(OH)D concentrations. Finally, residual confounding might still exist, though we tried to control several potential covariates.

## Conclusions

This retrospective cohort study showed the beneficial associations of early gestational vitamin D with outcomes of GDM, cesarean section, preterm birth, and low birth weight. More well-designed randomized clinical trials are needed for further exploration and confirmation of these results.

## Data Availability

The datasets used and/or analyzed during the current study are available from the corresponding author upon reasonable request.

## Abbreviations

GDM: Gestational diabetes mellitus
LBW: Low birth weight
BMI: Body mass index
SD: Standard deviation
